# Supplementary material for: Cardiac mitochondrial function depends on BUD23 mediated ribosome programming
Source: eLife. 2020 Jan 15;9:e50705. doi: 10.7554/eLife.50705 (PMC7002040; doi:10.7554/eLife.50705)
Supplement: Figure 6—source data 1. — LW/BW, lung weight/body weight; IVSD, interventricular septum thickness in diastole; IVSS, interventricular septum thickness in systole; LVPWD, posterior wall thickness in diastole; LVPWS, posterior wall thickness in systole; BPM, beat/per minute; RWT, relative wall thickness. Statistical p-values were defined using Student’s T-test. [file elife-50705-fig6-data1.docx]

**Echocardiogram analysis**

|  | Control  (n=10) | BUD23fl/fl MCK-Cre+/- (n=10) | P value |
| --- | --- | --- | --- |
| Body weight (BW), g | 9.97±0.44 | 11.04±0.74 | 0.232 |
| Heart weight (HW), mg | 63.04±2.95 | 60.95±4.05 | 0.682 |
| HW/BW ratio, mg/g | 6.33±0.16 | 5.63±0.38 | 0.108 |
| LW/ BW ratio, mg/g | 9.28±0.34 | 8.39±0.23 | 0.043 |
| IVSD, mm | 0.55±0.02 | 0.52±0.02 | 0.283 |
| IVSS, mm | 1.02±0.04 | 0.87±0.04 | 0.011 |
| LVPWD, mm | 0.60±0.05 | 0.54±0.03 | 0.314 |
| LVPWS, mm | 0.98±0.05 | 0.79±0.05 | 0.011 |
| Heart rate, BPM | 436±12 | 406±18 | 0.188 |
| RWT | 0.41±0.03 | 0.31±0.01 | 0.006 |

LW/BW, lung weight/body weight; IVSD, interventricular septum thickness in diastole; IVSS, interventricular septum thickness in systole; LVPWD, posterior wall thickness in diastole; LVPWS, posterior wall thickness in systole; BPM, beat/per minute; RWT, relative wall thickness. Statistical p-values were defined using Student’s T-test.

**Electrocardiogram analysis**

|  | Control  (n=10) | BUD23fl/fl MCK-Cre+/- (n=10) | P value |
| --- | --- | --- | --- |
| RR Interval (ms) | 151.34±7.39 | 160.15±8.28 | 0.438 |
| Heart Rate (BPM) | 404±18 | 384±19 | 0.463 |
| PR Interval (ms) | 43.72±1.53 | 42.54±1.68 | 0.610 |
| P Duration (ms) | 13.39±1.45 | 10.11±0.65 | 0.055 |
| QT Interval (ms) | 30.95±2.72 | 23.82±1.12 | 0.026 |

Statistical p-values were defined using Student’s T-test.
